# Molecular genotype in migraine

**DOI:** 10.1186/1129-2377-16-S1-A30

**Published:** 2015-09-28

**Authors:** Cherubino Di Lorenzo, Gaetano S Grieco, Anna Rubegni, Filippo M Santorelli

**Affiliations:** Don Gnocchi Foundation, Milan, Italy; Laboratory of Neurogenetics, C. Mondino National Institute of Neurology Foundation, IRCCS, Pavia, Italy; Molecular Medicine, IRCCS Stella Maris, Pisa, Italy

Migraine is an episodic brain disorder with disabling attacks of headache that are associated with nausea, vomiting, and hypersensitivity to light, sound, and smell. According to major criteria of the International Classification of Headache Disorders (ICHD-II) from the International Headache Society (IHS), migraine is divided into two main subtypes that are based on the absence (migraine without aura, MO) or presence (migraine with aura, MA) of an aura. Migraine has a profound effect on wellbeing and general functioning, not only during attacks, but also in terms of work performance, family and social relationships, and, mainly in children, school achievement, thus explaining why the WHO expert panel rates migraine among the most disabling and costly chronic disorders.

There is a strong genetic component in migraine as evidenced by observations that the disorder runs in families and that about 50% of the patients have close relatives also affected by a similar condition. However, migraine risk is also conferred by environmental factors and epidemiological evidence suggesting a tight gene-environment interaction (endogenous or exogenous), among which several predisposing or triggering factors have been defined.

In the past decades, our growing understanding of the genetic contributions in migraine disorders has been translated in better knowledge of the pathophysiology but needs to grow further and to be translated into more effective treatments. Indeed, several genes involved in syndromic and monogenic forms of migraine have been defined, allowing a significant contribution to the mechanisms generating the attacks, but the timing and specific contribution of secondary hits remain largely unclear.

